# Supplementary material for: Empirical methods for controlling false positives and estimating confidence in ChIP-Seq peaks
Source: BMC Bioinformatics. 2008 Dec 5;9:523. doi: 10.1186/1471-2105-9-523 (PMC2628906; doi:10.1186/1471-2105-9-523)
Supplement: Additional File 1 — A variety of html documents from the USeq web site detailing the available applications, their best usage, output file type descriptions, command line menus, etc. [file 1471-2105-9-523-S1.zip › USeqUserGuides/JavaDoc.zip!/JavaDoc/expr/ParseMiRNATable.html]

ParseMiRNATable


|  |  |  |
| --- | --- | --- |
| **Overview** \| **Package** \| **Class** \| **Use** \| **Tree** \| **Deprecated** \| **Index** \| **Help** | |  |
| **PREV CLASS**   **NEXT CLASS** | **FRAMES**    **NO FRAMES**     **All Classes** |  |
| SUMMARY: NESTED \| FIELD \| CONSTR \| METHOD | DETAIL: FIELD \| CONSTR \| METHOD |  |


---


## expr Class ParseMiRNATable

```
java.lang.Object
  expr.ParseMiRNATable
```

---

``` public class ParseMiRNATable extends java.lang.Object ```

---

**Constructor Summary**

`ParseMiRNATable(java.lang.String[] args)`


| **Method Summary** | |
| --- | --- |
| `static java.util.HashMap` | `hashMiRNATable(java.io.File txtTable)` |
| `static void` | `main(java.lang.String[] args)` |
| `static MiRNA[]` | `makeMiRNAs(java.util.HashMap map)` |
| `void` | `parseMiRNATable()` |
| `void` | `sumTargetScores()` |

**Methods inherited from class java.lang.Object**

`equals, getClass, hashCode, notify, notifyAll, toString, wait, wait, wait`

**Constructor Detail**

### ParseMiRNATable

```
public ParseMiRNATable(java.lang.String[] args)
```


**Method Detail**

### sumTargetScores

```
public void sumTargetScores()
```

---


### parseMiRNATable

```
public void parseMiRNATable()
```

---


### makeMiRNAs

```
public static MiRNA[] makeMiRNAs(java.util.HashMap map)
```

---


### hashMiRNATable

```
public static java.util.HashMap hashMiRNATable(java.io.File txtTable)
```

---


### main

```
public static void main(java.lang.String[] args)
```


---


|  |  |  |
| --- | --- | --- |
| **Overview** \| **Package** \| **Class** \| **Use** \| **Tree** \| **Deprecated** \| **Index** \| **Help** | |  |
| **PREV CLASS**   **NEXT CLASS** | **FRAMES**    **NO FRAMES**     **All Classes** |  |
| SUMMARY: NESTED \| FIELD \| CONSTR \| METHOD | DETAIL: FIELD \| CONSTR \| METHOD |  |


---
